# Dietary Outcomes of a Multilevel, Multicomponent, Cluster Randomized Obesity Intervention in 6 Native American Communities in the Upper Midwest and Southwest United States

**DOI:** 10.1016/j.cdnut.2023.100043

**Published:** 2023-02-06

**Authors:** Leslie C. Redmond, Caroline R. Wensel, Michelle Estradé, Sheila E. Fleischhacker, Lisa Poirer, Brittany Wenniserí iostha Jock, Joel Gittelsohn

**Affiliations:** 1 Department of International Health, Bloomberg School of Public Health, the Johns Hopkins University, Baltimore, MD, USA; 2 Department of Food and Human Nutritional Science, Faculty of Agricultural and Food Sciences, University of Manitoba, Ellis Building 242, 13 Freedman Crescent, Winnipeg, MB, Canada; 3 School of Medicine, the Johns Hopkins University, Baltimore, MD; 4 Georgetown University Law Center, Washington, DC, USA; 5 School of Human Nutrition, Center for Indigenous Peoples’ Nutrition and Environment, McGill University, Ste-Anne-de-Bellevue, Québec, Canada

**Keywords:** intervention, health promotion, health disparity, Native American, nutrition, dietary intake

## Abstract

**Background:**

Impacts of colonization on dietary intake have led to high rates of obesity and noncommunicable diseases among Native American adults. Multilevel, multicomponent (MLMC) interventions may improve dietary intake.

**Objectives:**

To assess the impact of a MLMC obesity intervention, OPREVENT2 (Obesity Prevention and Evaluation of InterVention Effectiveness in NaTive North Americans 2; clinicaltrials.gov NCT02803853), on dietary intake in Native American adults in Intervention versus Comparison communities.

**Methods:**

A cluster-randomized controlled trial was performed among participants in 6 communities randomized to Intervention (*n =* 3 and Comparison (*n =* 3). Adults aged 18 to 75 were recruited from tribal communities in the Southwest and upper Midwest United States from September 2016 to May 2017 (*n =* 601). This analysis included participants who completed baseline and follow-up surveys (82% retention), reported dietary intake between 500 and 7000 kcal/d, and had no missing data for outcomes of interest (*n =* 446). The intervention was implemented from May 2017, to November 2018. OPREVENT2 integrated individual, environmental, social, and structural factors and was implemented in food stores, worksites, schools, and community media outlets in Intervention communities. Activities included taste tests, cooking demonstrations, and stocking healthier items in food stores and were reinforced by a social m)edia campaign, posters, brochures, and booklets focused on nutrition. Individual-level dietary intake among participating Native American adults was assessed via modified Block food-frequency questionnaire at preintervention and postintervention. Multilevel mixed-effects linear regression, with clustering at the community level, was performed.

**Results:**

Between-group effects were significant (*P* < 0.05) for intake of carbohydrates (-23 g/d), total fat (-9 g/d), saturated fats (-3 g/d), and monounsaturated fats (-4 g/d), with greater decreases in Intervention communities. Between-group effect for total sugar (-12 g/d in Intervention communities) was not statistically significant.

**Conclusions:**

This MLMC intervention was associated with significantly improved carbohydrate, total fat, and saturated fat intake among Native American adults. These changes are important for improving health within this population.

## Introduction

Many Native American precolonial dietary patterns—rich in fruits, vegetables, whole grains, nuts, fish, and lean wild game [[1], [2], [3]]—are considered health promoting and anti-inflammatory [[4], [5], [6]]. Conversely, colonization forced Native American communities to shift to a Westernized diet—high in saturated fat and refined carbohydrates—which is linked with high rates of obesity and noncommunicable diseases (NCDs) [[7], [8], [9]]. Today, Native Americans bear an inequitable burden of obesity and diet-related NCDs compared with other ethnicities in the US [[10], [11], [12]], and these disparities are largely attributable to the intergenerational trauma of genocidal policies and actions against Native Americans that resulted in the destruction of relationships with their homelands and foodways [2,3,13].

National efforts, such as the *Dietary Guidelines for Americans* (DGA), offer nutrition professionals and the public advice on how healthy Americans can meet nutrient needs and help prevent diet-related NCDs (e.g., minimizing intake of trans fats, limiting intake of saturated fats to less than 10% of total daily calories and replacing them with MUFAs and PUFAs, and limiting added sugars to less than 10% of total daily calories) [14]; however, these recommendations are difficult to follow when access to affordable, quality, nutritious foods and beverages is limited, as is the case for many Native American communities [2]. There has been a concerted effort to culturally adapt nutrition programs and interventions for Native American populations; however, few interventions have aimed to improve the food environment [13], a key determinant of diet-related health disparities [15]. Single component intervention programs targeting the retail food environment have had moderate success in overcoming barriers to health equity, but more work remains as Native American health disparities persist. For example, the Navajo Healthy Stores intervention combined environmental changes and nutrition education in local food stores to improve behavioral and health outcomes and showed that increased exposure to the intervention was associated with a reduction in BMI and improved healthy food intentions (16). Additionally, the THRIVE Study (Tribal Health and Resilience in Vulnerable Environments), which assessed healthy retail strategies in tribally owned stores, found that exposure to the intervention was associated with the purchase of healthier items but did not improve intake [17].

Compared with single component food store interventions, multilevel multicomponent (MLMC) interventions offer an innovative and promising way to address multiple aspects of the environment by incorporating numerous components that reinforce each other through several aspects of daily life [16]. To our knowledge, few previous studies have implemented a MLMC approach to address obesity in Indigenous communities [18]. Of these, most report on more distal factors, such as changes to food-related knowledge and self-efficacy, and few report on changes in individual-level dietary intake. One study, *Zhiiwaapenewin Akino’maagewin: Teaching to Prevent Diabetes,* took place in schools, food stores, and other community locations in 7 Northwestern Ontario First Nations in Canada and resulted in improved knowledge of healthier behaviors and healthy food acquisition among adults [19]. Healthy Foods North, an intervention in Indigenous communities in the Canadian Arctic that took place in local food stores, recreation centers, and schools, improved participants’ food-related self-efficacy and intentions [20]. Finally, the first OPREVENT trial, which was conducted in local food stores, worksites, and schools, led to a significant decrease in regular, sugar-sweetened soda consumption but not other discouraged sugary beverages [21]. Despite these positive findings, evidence of MLMC intervention impacts on more proximal factors such as diet quality and dietary intake as indicated by the Healthy Eating Index (HEI) 2015 [22], energy, macronutrient, and food group consumption remains, to the authors’ knowledge, underreported.

The current trial, OPREVENT2, expanded the scope of OPREVENT and took place in local food stores, worksites, schools, and other venues specific to individual communities such as health clinics and community centers, and incorporated institutional policy [23]. The purpose of this analysis was to examine the individual-level impact of the OPREVENT2 trial on the dietary intake of intervention participants, with special attention to the different types of dietary fat and carbohydrates. It was hypothesized that participants in Intervention communities would have improved diet quality and dietary intake compared with participants in Comparison communities.

## Methods

### Study design

OPREVENT2 was a community-randomized controlled MLMC obesity prevention trial conducted in 6 Native American communities in the upper Midwest and Southwest United States. The primary outcome was BMI. The objective of this analysis was to report on individual-level dietary intake.

After preintervention data collection, 3 communities were randomized to receive the OPREVENT2 intervention immediately via hat draw, which was identified by participating communities as a culturally appropriate approach to randomization. Tribal leaders in Midwestern communities drew names from a hat for Intervention communities in the Southwest, and tribal leaders in the Southwestern communities drew names from the hat for Intervention communities in the Midwest. Postintervention data collection then took place in all 6 communities from December 2018 to August 2019, after which the 3 wait-listed Comparison communities received the intervention for purposes of equity (no further data collection occurred because of budgetary constraints).

The OPREVENT2 program and evaluation received Institutional Review Board (IRB) approval from the Johns Hopkins Bloomberg School of Public Health IRB, the Navajo Nation Human Research Review Board, and the Indian Health Service IRB. Approval was also received from individual tribal councils. This trial was funded by the National Institutes of Health National Heart, Lung, and Blood Institute (R01HL122150) and registered with clinicaltrials.gov (NCT02803853).

### Participants and recruitment

Before submitting the grant that would fund OPREVENT2, the research team was in contact with 37 interested tribal communities in the Southwest and Midwest United States. Of those, 4 in the Southwest and 2 in the Midwest followed through with letters of support and memoranda of understanding in support of the OPREVENT2 grant application. Subsequent to receiving funding, these 6 communities passed tribal resolutions to affirm their intentions to collaborate with the research team in carrying out the OPREVENT2 project. These written agreements between the tribes and research team included plans for tribal ownership of the data as well as a tribal approval process for publishing and presenting results of the research. Further details of tribal eligibility and engagement have been published elsewhere [23, 24].

Approximately 100 adults from each community (*n =* 601) were recruited from September 2016 to May 2017 to be part of the preintervention sample via data collectors calling randomly selected participants from a contact list provided by the tribal governments and through community media. Participants were eligible to participate if they were not pregnant or breastfeeding, were between the ages of 18 and 75, self-identified as current tribal members within the community, and planned to stay in the community for at least the next 2 y. Of the 859 adults screened to participate, 27.2% were ineligible because of not meeting all inclusion criteria and 2.8% refused. Signed informed consent was obtained from all eligible respondents who agreed to participate. Consent included that all data was to be deidentified and that compensation would be provided in the form of gift cards ($40) for each meeting, regardless of whether the participant completed the study. Individuals were informed that there was no expected direct benefit to participating but that the information collected would be used to develop a program to help improve the diet and exercise of people in their community. Memoranda of understanding between each participating community and the Johns Hopkins University study team discussed the reporting and approval of data in manuscripts, presentations, and other documents such as progress reports. Study progress reports (2–5/y) were made to tribal councils and/or health committees as oral presentations and/or written reports.

### Intervention design

The theoretical framework for the OPREVENT2 intervention was based on Bandura’s Social Cognitive Theory, which views psychosocial factors such as self-efficacy, outcome expectations, and self-regulation, situated within a broader social context, as elements of the causal pathway to individual behavior change [25]. It also drew from Bronfenbrenner’s Social Ecological Model, which views different levels of society as a system of reciprocal influences that can shape and be shaped by one another [26]. During an extensive formative phase of research, the intervention was designed to address unique barriers to healthy dietary and physical activity behaviors in rural reservation communities [23, 24]. The MLMC intervention included institutional-level components (food stores, worksites, schools, Community Action Committees), interactive experiences (taste tests, cooking demonstrations, giveaways), and distribution of educational media/materials (community newsletters, posters, booklets, social media, radio announcements). OPREVENT2 was delivered in 6 phases, each lasting 2–4 mo, over a total intervention period of 18 mo.

The design and implementation of the nutrition component of the OPREVENT2 intervention was overseen by 2 registered dietitian nutritionists (RDNs) and a nutritional anthropologist (study principal investigator [PI]) with continuous tribal community partnership. Trainings were conducted with a team of 7 data collectors and interventionists, which included both Native American and non-Native American graduate students and individuals hired from within the tribal communities; the RDNs provided training related to anthropometric measurements and nutritional content, and the nutritional anthropologist provided additional training on communication strategies and cultural sensitivity.

The primary dietary targets of the intervention were to decrease total energy, fat, and sugar intake by choosing lean proteins, whole grains, low-fat dairy, fruits, and vegetables. Each phase focused on a specific educational message or goal to align with these targets, such as decreasing saturated fat, increasing dietary fiber, reducing added sugars, and selecting lean proteins. Before intervention implementation, research staff including the PI, RDNs, graduate students, and community member hires worked to identify healthier, lower in fat and added sugar options already available in each community. During the intervention, they worked with local food store owners to promote and improve access to these items. Interventionists conducted cooking demonstrations and taste tests in local food stores and checked weekly to ensure that healthy foods and beverages were stocked and healthy options were highlighted using shelf labels. Examples of promoted foods included traditional and nontraditional foods (e.g., salmon, raw seeds and nuts, wild rice, low-fat yogurt, beans, apples, berries, celery, salad greens). Ultraprocessed foods (e.g., soda, potato chips, cookies, donuts, sweetened breakfast cereals) were discouraged. At least 1 worksite in each intervention community offered regular physical activity opportunities for employees, such as pedometer challenges or onsite workout equipment, and at least 1 elementary school per community implemented a curriculum that taught children in grades 2 through 6 about traditional healthy foods and practices. The school curriculum did not target child behavior, but instead was envisioned as a way to help children act as change agents in their households, in that they would support and motivate adult behavior change in their families and wider social networks [27]. Posters, booklets, newsletters, social media posts, and radio announcements were widely distributed in intervention communities to reinforce the key educational messages during each phase of the intervention.

### Instruments

To assess dietary intake, a semiquantitative Block FFQ was adapted from one previously validated and used in the Strong Heart Study [28]. Use of FFQs to assess dietary intake and impact of community-based nutrition interventions in Native American communities is documented in the literature [[29], [30], [31]], and FFQs can provide valid, reliable assessments of energy intake, macronutrient intake, and dietary patterns as defined by food groups [32]. During the formative phase of research, questions about foods of regional and cultural relevance were added, such as mutton and venison. The FFQ used in this analysis probed the frequency of intake for 113 foods over the previous 30 days. It was pilot tested in 2 tribal communities that did not participate in OPREVENT2 but were located near participating communities. The purpose of the pilot test was only to determine whether community members felt that the FFQ adequately captured foods of regional and cultural relevance as determined in the formative phase and to ensure that the FFQ instructions, questions, and response options were easily understandable. This was accomplished by administering the survey in a small sample of community members and following up with a short, informal oral conversation to ensure that it was done in a culturally sensitive way. After the pilot testing, feedback was positive and did not indicate necessary changes, therefore no edits to the FFQ were made.

An Adult Impact Questionnaire was used to record demographics and anthropometric measurements. Demographic data included age, sex, marital status, employment status, highest level of education, household size, and smoking status. As a proxy for socioeconomic status, a Material Style of Life (MSL) score was calculated from an additive index of 18 questions about the number of material possessions belonging to the household (see [33] and [19] for details on MSL). This method was selected as a more culturally acceptable alternative to asking direct income-related questions.

Intervention implementation was assessed with food store, Worksite, School, Social Media, and Community Action Committee Impact Questionnaires, which were designed to measure reach, dose delivered, and fidelity of each component. Reach was defined as the number of individuals in the intended priority audience who participated at any level in the intervention component and was measured at the individual and institutional levels. Dose was defined as the number of units of each intervention component provided by OPREVENT2 interventionists. Fidelity was assessed based on reactions to or level of engagement with a program component, such as number of likes on a Facebook post or stocking of promoted foods in stores. Process data were not included in this analysis but have been reported elsewhere [34].

All data collectors, many of whom were hired from the participating communities, participated in a weeklong in-person training before beginning the preintervention data collection, as well as a 2-d booster training before postintervention data collection. Data collectors were highly familiar with the cultures and languages of the communities in which they collected data and translated the consent forms and interview questionnaires into the local languages as necessary and if requested by a participant to ensure understanding. All data collection took place during in-person, one-on-one interviews in easily accessible community spaces that also offered participant privacy.

### Outcomes

Individual-level outcome measures for this analysis included change in HEI-2015 score and change in daily energy and macronutrient intake, both in total grams and percent total daily calories, from preintervention to postintervention assessed using the FFQ. Additional individual-level outcome measures included change in daily intake of types of dietary fats (including saturated fat, MUFAs, PUFAs, trans fats, and cholesterol), types of carbohydrates (including dietary fiber and total sugar), and daily servings of food groups as defined by the 2015–2020 DGA: vegetables, fruits, grains, protein foods, dairy, and fats and oils/sweets [14], plus servings of regular sugar-sweetened soda. Change values for each primary and secondary outcome were calculated by subtracting the preintervention value from the postintervention value. These change values were used as the dependent variables for this analysis.

### Data management

The PI and data manager were responsible for the security of identifiable data. Data from the Adult Impact Questionnaire were entered into Microsoft Access databases and Microsoft Excel spreadsheets (Microsoft Corporation), then exported to Stata software, version 16 [35] for analysis. All completed FFQs were sent to NutritionQuest [36] for processing and calculation of nutrient intakes. Results were sent back to the research team as a Microsoft Excel spreadsheet and exported to Stata.

### Statistical analysis

Sample size was determined based on BMI impact data from the first OPREVENT trial for an effect size of 1.3 kg/m^2^, a type I error of 5%, and an intraclass correlation coefficient of 0.0001, leading to a power of about 0.8 with 80 participants per community, or a total sample size of 480 [23]. Comparisons of preintervention characteristics between Intervention and Comparison communities, as well as between participants who completed data collection at both time points and those who did not to explore patterns of missingness, were conducted using Student *t*-tests for normally distributed continuous variables, nonparametric Wilcoxon-Mann-Whitney tests for nonnormal continuous variables, and chi-square tests for categorical and dichotomous variables.

To assess intervention impact, between-group effects were calculated using multilevel mixed-effects linear regression, with clustering at the community level. These are reported as regression coefficients (β) with confidence intervals. Within-group effects were also considered and calculated using multilevel mixed-effects linear regression, with clustering at the community level. Within-group effects are reported as regression coefficients (β) with confidence intervals. All models were adjusted for a priori and statistically determined covariates. These included age, sex, education, employment, MSL score, smoking, and the preintervention dependent variable of interest. Statistical significance was set, a priori, at an alpha level of <0.05.

## Results

**FIGURE 1:**
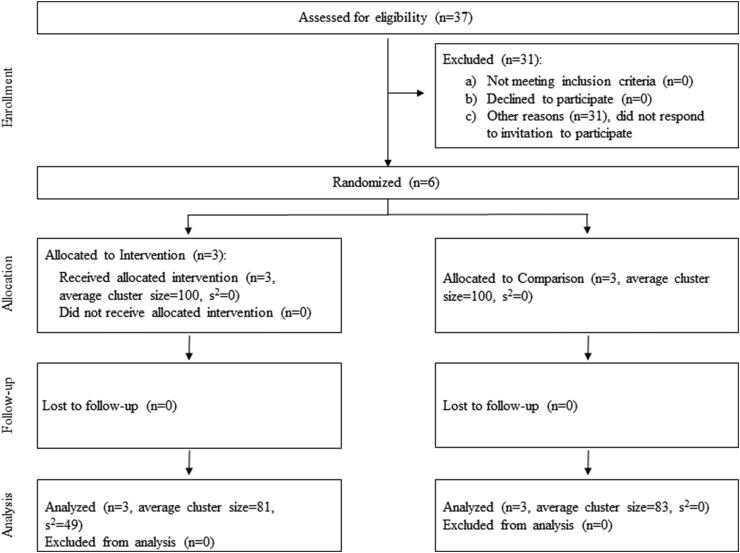
OPREVENT 2 community enrollment and randomization

**FIGURE 2:**
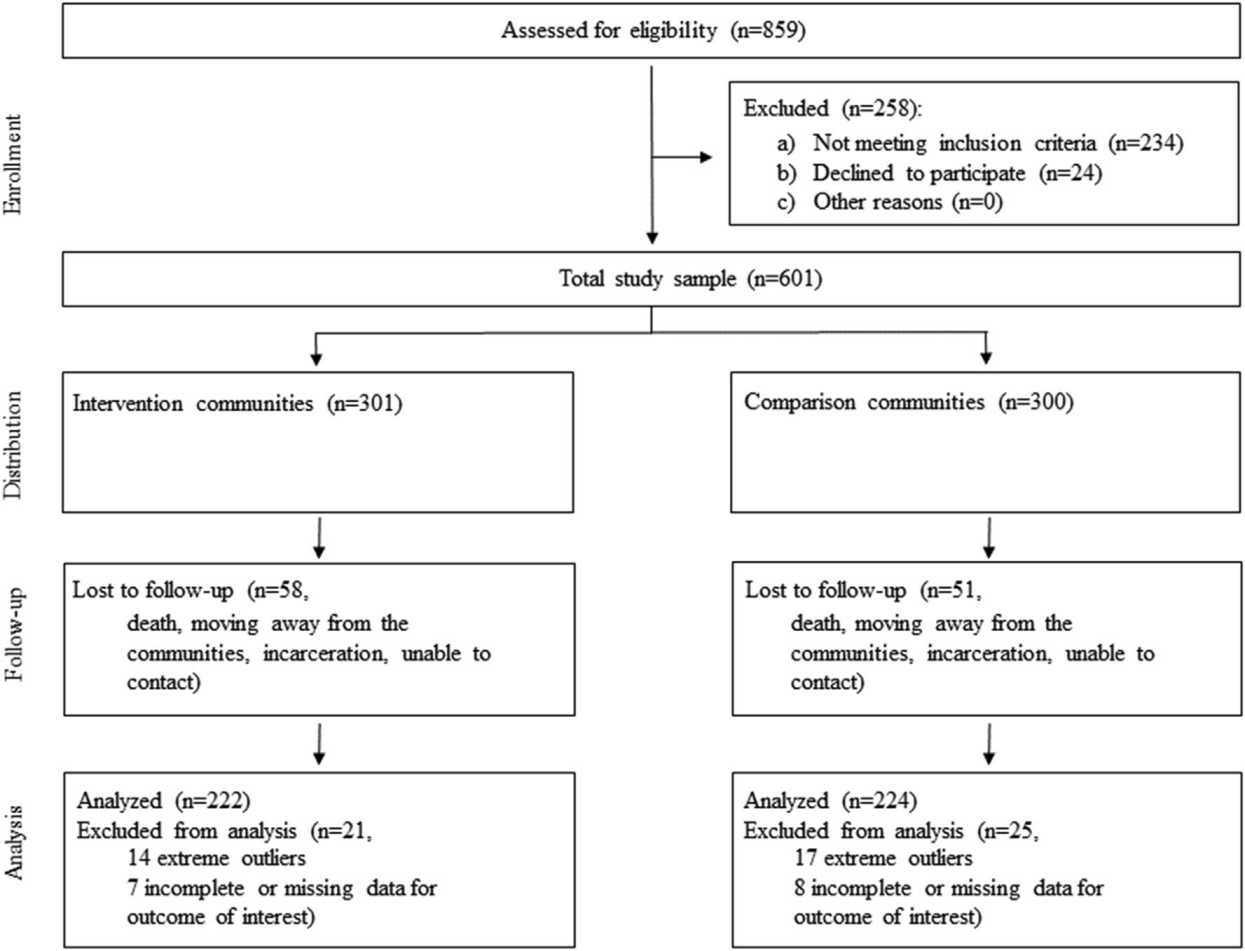
OPREVENT 2 participant enrollment and randomization

Community enrollment and randomization is shown in Figure 1. Of the 37 communities invited to participate, 31 were excluded and 6 were randomized to Intervention (*n =* 3) or Comparison (*n =* 3); all 6 were included in analysis. Participant enrollment and randomization is shown in Figure 2. Of the 601 participants who completed dietary surveys preintervention, 301 were located in Intervention communities and 300 were located in Comparison communities. A total of 492 also completed postintervention surveys (82% retention rate; *n =* 243 in Intervention and *n =* 249 in Comparison), with 109 participants lost to follow-up across all communities for reasons including death, moving away from the communities, incarceration, and inability to contact. Participants reporting daily caloric intake greater than 7000 kcal or less than 500 kcal were excluded from analysis (*n =* 14 in Intervention and *n =* 17 in Comparison), as these values were identified as extreme outliers [37,38]. Of the remaining total 461 participants with pre- and postintervention data, 15 had incomplete or missing data for outcomes of interest (*n =* 7 in Intervention and *n =* 8 in Comparison) and were also excluded from the analysis, for a final analysis sample of 446 participants, with 222 located in Intervention communities and 224 located in Comparison communities.

The analysis did not reveal statistically significant differences in preintervention characteristics between participants who completed data collection at both time points and those who did not (data not shown). Preintervention demographic characteristics of the final analysis sample (*n =* 446) are shown in Table 1. Participants were predominantly female (71%) with an average age of 47 y. Nearly 40% of participants had completed some college, and 42% were employed full time. Average BMI was just over 31 kg/m^2^. There were statistically significant differences in mean age, education, MSL score, and employment status between Intervention and Comparison communities (*P* < 0.01).

**TABLE 1:** Comparison of baseline sociodemographic characteristics of the OPREVENT2 analysis sample^1^

	Analysis Sample (*n =* 446)	Comparison (*n =* 224)	Intervention (*n =* 222)	*P*
Age, y	47.15 **±** 14.85	49.58 **±** 15.18	44.71 **±** 14.12	< 0.01∗
Female	316 (71)	155 (70)	161 (73)	
Education				<0.01∗
Less than high school	80 (18)	54 (24)	26 (12)	
High school or GED	165 (37)	96 (43)	69 (31)	
Some college	175 (39)	65 (29)	110 (50)	
College or more	24 (5)	8 (4)	16 (7)	
MSL Score	14.06 **±** 8.10	11.86 **±** 7.86	16.28 **±** 7.74	<0.01∗
Marital Status				0.12
Single/Never Married	173 (39)	81 (36)	92 (42)	
Married	140 (31)	33 (70)	67 (31)	
Separated	13 (2)	7 (3)	6 (3)	
Divorced	47 (10)	17 (8)	30 (14)	
Other^2^	66 (15)	42 (19)	24 (11)	
Employment Status				<0.01∗
Full time	187 (42)	54 (24)	133 (60)	
Part time	58 (13)	38 (17)	20 (9)	
Retired	36 (8)	30 (13)	6 (3)	
Disabled	19 (4)	14 (6)	5 (2)	
Other^3^	45 (10)	26 (12)	19 (9)	
Smokes Commercial Tobacco	127 (29)	63 (28)	64 (29)	0.87
BMI (kg/m^2^)	31.79 **±** 6.64	31.52 **±** 6.47	32.08 **±** 6.81	0.38

1 Values are mean **±** standard deviation or *n* (%); independent Student *t*-tests for normally distributed continuous variables, nonparametric Wilcoxon-Mann-Whitney tests for nonnormal continuous variables, and chi-square tests for categorical and dichotomous variables; ‘∗’ denotes significantly different at the *P* < 0.05 level; GED, general educational development; MSL, Material Style of Life; OPREVENT2, Obesity Prevention and Evaluation of InterVention Effectiveness in NaTive North Americans 2

2 Common law, lives with partner

3 Seasonal/temporary, student, self-employed

### Change in daily energy intake, macronutrients, and percent daily calories from macronutrients

Table 2 shows change in daily energy intake and macronutrients as well as percent of daily calories from each macronutrient for both Comparison and Intervention communities from preintervention to postintervention. The between-group effect comparing Intervention and Comparison communities was statistically significant for daily intake of carbohydrate (-23 g; [-45, - 0]) and total fat (-9 g; [-17, - 0]). Between-group effects were not statistically significant for daily energy or percent daily calories from any of the macronutrients.

**TABLE 2:** Within and between-group effects for diet quality and dietary intake of Native American adults participating in the OPREVENT2 evaluation^1^

	Comparison Communities (*n* = 224)			Intervention Communities (*n* = 222)			Between-Group Effect (95% CI)
	Preintervention (mean ± SD)	Posintervention (mean ± SD)	Within-Group Effect (95% CI)	Preintervention (mean ± SD)	Postintervention (mean ± SD)	Within-Group Effect (95% CI)	
*Change in Daily Energy Intake, Macronutrients, and Percent Daily Calories from Macronutrients*							
Energy (kcal)	2222 **±** 1177	2134 **±** 1112	-88 (-299 – 124)	2070 **±** 1056	1914 **±** 973	-156 (-343 – 30)	-188 (-377 – 0)
Carbohydrates (g)	254 **±** 144	243 **±** 131	-11 (-36 – 15)	240 **±** 128	218 **±** 115	-23 (-45 – 0)∗	-23 (-45 – 0)∗
Protein (g)	86 **±** 51	82 **±** 47	-4 (-13 – 5)	789 **±** 44	73 **±** 39	-5 (-13 – 2)	-6 (-14 – 1)
Fat (g)	97 **±** 51	93 ± 50	-4 (-13 – 6)	89 **±** 48	83 **±** 44	-6 (-15 – 2)	-9 (-17 – 0)∗
% Daily Calories Carbohydrates	45 **±** 7	46 **±** 7	0 (-1 – 2)	46 **±** 7	45 **±** 8	-1 (-2 – 1)	0 (-2 – 1)
% Daily Calories Protein	16 **±** 3	15 **±** 3	0 (-1 – 1)	15 **±** 3.0	16 **±** 3	0 (0 – 1)	0 (0 – 1)
% Daily Calories Fat	40 **±** 6	40 **±** 6	0 (-1 – 1)	39 **±** 6	39 **±** 6	0 (-1 – 1)	0 (-2 – 1)
*Change in Daily Intake of Total Sugar, Whole Grains, and Fiber*							
Total Sugar (g)	111 **±** 72	108 **±** 70	-3 (-16 – 10)	110 **±** 67	99 **±** 62	-10 (-22 – 1)	-12 (-25 – 0)
Whole Grains (g)	0 **±** 0	0 **±** 0	0 (0 – 0)	0 **±** 0	0 **±** 0	0 (0 – 0)	0 (0 – 0)
Fiber (g)	16n **±** 10	15 **±** 8	-1 (-3 – 1)	15 **±** 8	14 **±** 7	-1 (-3 – 0)	-1 (-3 – 0)
*Change in Daily Intake of Saturated Fat, MUFAs, PUFAs, Trans Fats, and Cholesterol*							
Saturated Fat (g)	32 **±** 18	31 **±** 17	-1 (-4 – 2)	29 **±** 16	27 **±** 15	-2 (-5 – 1)	-3 (-6 – 0)∗
MUFAs (g)	39 **±** 21	38 **±** 20	-2 (-5 – 2)	36 **±** 20	34 **±** 18	-3 (-6 – 1)	-4 (-7 – 0)∗
PUFAs (g)	18 **±** 10	18 **±** 10	-1 (-2 – 1)	17 **±** 10	16 **±** 9	-1. (-3 – 0)	-2 (-3 – 0)
Trans Fats (g)	3 **±** 2	3 **±** 2	0 (-1 – 0)	3 **±** 2	3 **±** 2	0 (-1 – 0)	0 (-1 – 0)
*Change in Daily Servings of Food Groups and Soda*^2^							
Servings Vegetables	3 **±** 2	3 **±** 2	0 (-1 – 0)	2 **±** 2	2 **±** 2	0 (0 – 0)	0 (-1 – 0)
Servings Fruits	1 **±** 1	1 **±** 1	0 (0 – 0)	1 **±** 1	1 **±** 0	0 (0 – 0)	0 (0 – 0)
Servings Grains	7 **±** 5	7 **±** 4	0 (-1 – 1)	6 **±** 4	6 **±** 3	-1 (-1 – 0)	0 (-1 – 0)
Servings Protein	3 **±** 2	3 ± 2	0 (0 – 0)	3 **±** 2	3 **±** 2	0 (0 – 0)	0 (-1 – 0)
Servings Dairy	1 **±** 1	1 **±** 1	0 (0 – 0)	1 **±** 1	1 **±** 1	0 (0 – 0)	0 (0 – 0)
Servings Fats & Sweets	4 **±** 2	4 **±** 2	0 (-1 – 0)	4 **±** 2	4 **±** 2	0 (-1 – 0)∗	0 (-1 – 0)∗
Servings Sodas	0 **±** 0	0 **±** 0	0 (0 – 0)	0 **±** 0	0 **±** 0	0 (0 – 0)	0 (0 – 0)
*Change in Healthy Eating Index (HEI) 2015*^3^							
HEI	49 ± 8	49 ± 7	0 (-2 – 1)	50 ± 34	50 ± 8	0 (-1 – 2)	1 (-1 – 3)

1 Within-group effects represent the difference in outcomes from preintervention to postintervention for Comparison and Intervention communities; between-group effects represent the differences in magnitude of change between Comparison and Intervention communities; results are from multilevel mixed-effects linear regression models and reported as regression coefficients (β) with confidence intervals; models were adjusted for a priori and statistically determined associations including age, sex, education, employment, Material Style of Life (MSL) score, smoking, and the baseline dependent variable of interest; ‘∗’ denotes significantly different at the *P* < 0.05 level; CI, confidence interval; OPREVENT, Obesity Prevention and Evaluation of InterVention Effectiveness in NaTive North Americans

2 Food groups defined by the Dietary Guidelines for Americans 2015-2020 as follows: vegetables (all starchy and non-starchy); fruits (fruits and fruit juices); grains (breads, cereals, rice, pasta); protein (meat, fish, poultry, beans, eggs); dairy (milk, yogurt, cheese); fats & sweets (fats, oils, sweets, sodas) (14); servings sodas included regular, sugar-sweetened sodas

3 Healthy Eating Index 2015: measure of diet quality used to assess how well a set of foods aligns with key recommendations of the Dietary Guidelines for Americans, scored 0–100 (52)

### Change in daily intake of total sugar, whole grains, and fiber

There was modest impact to daily intake of total sugar, whole grains, and fiber for Comparison and Intervention communities. The between-group effects were not statistically significant for any of these outcomes.

### Change in daily intake of saturated fat, MUFAs, PUFAs, trans fats, and cholesterol

Only the between-group effects for daily intake of saturated fat (-3 g; [-6, - 0]) and MUFAs (-4 g; [-7, - 0]) were statistically significant.

### Change in daily servings of food groups

There were no changes to daily servings of any food groups. Despite statistical significance in daily servings of fats and sweets, the change was not meaningful.

### Change in Healthy Eating Index

The intervention did not have a measurable impact on the HEI-2015 score.

## Discussion

To the authors’ knowledge, this is the first MLMC trial among Native American adults to show significant impacts on dietary intake. Additionally, several changes with practical importance and potential positive impact to health were also observed. Daily intake of carbohydrate, total fat, saturated fat, and MUFAs showed statistically significant decreases in the Intervention communities compared with the Comparison communities. Daily energy and total sugar intake also decreased. Although these decreases did not reach statistical significance, a daily decrease of 188 kcal and 12 g of sugar may still result in meaningful changes to health outcomes, such as helping to manage blood glucose levels or making it easier to achieve a healthy weight. These results are promising, as the OPREVENT2 intervention messages and activities specifically targeted saturated fat, added sugars, and other refined carbohydrates. However, overall diet quality as measured by the HEI-2015 was not impacted, and there were some targeted nutrients and food groups that remained unchanged or without meaningful change, such as whole grains, dietary fiber, and daily servings from all food groups.

The 2015–2020 DGA recommend a daily energy intake of 2000 kcal/d for a moderately active adult [14]. Mean daily energy intake for both Comparison and Intervention communities of the OPREVENT2 analysis sample was close to this recommendation at both pre- and postintervention.

For the entire sample, average percent of daily energy intake from carbohydrates and protein were within the Acceptable Macronutrient Distribution Range at both pre- and postintervention. Conversely, the average percent of daily energy intake from fats exceeded the upper limit of the Acceptable Macronutrient Distribution Range in the entire sample at both pre- and postintervention. Further analysis showed that the average percent of daily energy intake from saturated fat was also above the recommendation. This is concerning, as intake of saturated fat has been linked to increased risk of chronic disease mortality [39].

Given that daily total fat and saturated fat intake were both well above what is recommended, it is especially noteworthy that the OPREVENT2 intervention resulted in statistically significant decreases in daily intake of both total fat (-9 g; [-17, - 0]) and saturated fat intake (-3 g; [-6, - 0]). Although percent calories from fat still remained above the recommendation at postintervention, this decreased intake is encouraging and shows that much of the decrease in total fat can be attributed to a decrease in saturated fat, which aligns with the 2015–2020 DGA recommendations. This change also demonstrates practical importance, as 9 g of fat is nearly 2 standard 5-g servings of fat and represents a daily decrease of 81 kcal, which may contribute to overall calorie reduction and achievement of a healthy weight. Intervention messaging promoted choosing lower-fat foods, and specifically, foods lower in saturated fat. Messages included shelf labels identifying lower-fat products and other written materials and taste tests highlighting lower-fat cooking methods, like using cooking spray and grilling or baking foods instead of frying them.

In addition to changes in total fat and saturated fat intake, the average decrease in daily intake of MUFAs was also statistically significant (-4 g; [-7, - 0]). Research on the impact of MUFAs on health outcomes such as cardiovascular disease, type 2 diabetes, and overall mortality is evolving. Consumption of dietary MUFAs has been associated with improved blood lipid profiles, blood pressure, insulin sensitivity, and blood glucose levels, as well as overall decreased risk of obesity [40]. Current evidence also suggests that MUFAs from plant-based sources provide protective benefits whereas MUFAs from animal-based sources may contribute to increased health risk [41, 42]. Although intervention messages did provide information on consuming healthy fats and foods that contain healthy fats, they did not distinguish between MUFAs and PUFAs. Future interventions should consider providing this information so that consumers can make informed choices to support their overall health.

Evidence on dietary fat’s health impacts among Native American populations is mixed. For example, consumption of full-fat dairy was associated with lower incidence of type 2 diabetes among Native Americans participating in the Strong Heart Family Study [43]. Of note, overall dairy food intake was low in the study population, and lactose intolerance is common among many Native American populations. Daily servings of dairy (including milk, yogurt, and cheese) in our sample were below the recommended 3 servings per day in Comparison and Intervention communities at both pre- and postintervention. Previous work conducted in an Indigenous Canadian population found that high consumption of foods in the “fat and butter food group” was associated with increased risk for diabetes [44] and that dietary patterns characterized by high-fat foods were associated with greater prevalence of cardiovascular disease and diabetes [45]. Dietary patterns characterized by high “junk food” intake, including processed and high-fat fried foods, have been associated with increased insulin resistance in Cree adults living in Northern Québec [46], and dietary patterns characterized by high intake of high-fat beef and processed foods have been linked to increased risk for incident type 2 diabetes among adults living in Indigenous communities in Canada [47]. A recent systematic review also identified diets high in fat and carbohydrates as a risk factor for obesity among Native American adults [12]. The average BMI of our sample was over 30 kg/m^2^ in both the comparison and intervention groups, indicating that identifying and mediating risk factors for obesity is important.

In addition to fat intake, carbohydrate intake also plays a critical role in the etiology of diet-related NCDs such as obesity and type 2 diabetes [48]. Like fats, it is important to distinguish between types of carbohydrates, as different types are associated with divergent health outcomes. The 2015–2020 DGA recommend limiting added sugars and refined starches while increasing consumption of whole grains and fiber, and specifically state that fewer than 10% of daily calories should come from added sugar while at least half of the grains consumed each day should be whole grains [14]. The 2015–2020 DGA also recommend that adults aged 31 to 50 should consume approximately 25–30 g of fiber per day. The OPREVENT2 intervention resulted in a statistically significant decrease in daily carbohydrate intake (-23 g; [-45, -0]). This represents 1.5 standard 15-g servings of carbohydrates, or about 92 kcal, and could contribute to overall calorie reduction and achievement of a healthy weight. The intervention did not result in a statistically significant increase in whole grain or fiber intake despite the availability of high fiber foods and focused intervention messaging, including shelf labels identifying foods with higher fiber content and other written materials communicating the health benefits of fiber.

While the FFQ did not allow for quantification of added sugars, estimates of total daily sugar intake and daily servings from the fats and sweets group were obtained, which included many items with added sugar (i.e., sweets, sodas). The OPREVENT2 program resulted in a decrease in total daily sugar intake (-12 g; [-25, - 0]), although this change was not statistically significant. Impact on change in daily servings of fats and sweets was statistically significant, decreasing by 0.4 servings per day. This is consistent with intervention messaging, which emphasized reducing sugar intake, specifically added sugars, and limiting sweets. Messages also focused heavily on reducing sugar-sweetened beverage intake; however, unlike in the first OPREVENT trial [21], no statistically significant change was observed in soda intake specifically. It should be noted that preintervention intake of soda in this sample was less than 1 serving per day in both Intervention and Comparison communities, so the lack of statistically significant change is not unexpected. Discussions on sugar-sweetened beverage taxes have gained momentum recently within several tribal communities, and although it is possible that this low reported intake of soda was related to the heightened awareness of and attention to sugar-sweetened beverage intake because of discussions or implementations of these tax initiatives, this study did not collect data that would allow for further investigation into this theory.

Whole grains and fiber have been shown to have protective roles against obesity, type 2 diabetes, and other diet-related chronic diseases. Conversely, added sugars and refined grains have been shown to contribute to the development of these conditions. Low-fiber diets have been associated with the development of type 2 diabetes in a remote Aboriginal population in Canada [49], and a study by Lazzinnaro and colleagues in 2012 found that percent of energy intake from carbohydrates (controlling for sugar intake) was inversely associated with BMI, but that daily intake of sugar-sweetened beverages was positively associated with BMI and impaired fasting glucose among a Canadian Indigenous community [50]. Unhealthy snack consumption, characterized by intake of foods like chocolate, candy, and chips, has also been associated with cardiovascular disease in Native American populations in Michigan and New Mexico [45].

Despite these many positive outcomes, there was no statistically significant change to the HEI-2015 score. There is a lack of data reporting on change in HEI-2015 or other diet quality indices in Native American populations as a result of interventions. Only one study was found to be similar, for which the Alternative Healthy Eating Index 2010 was used to assess the impact of a diabetes prevention and management program in Native American youth. Similar to the current analysis, authors reported improvements to individual measures of dietary intake, such as reduced daily energy intake, but found no change in the Alternative Healthy Eating Index 2010 score [51]. Higher scores on the HEI-2015 are known to be associated with reduced risk for cardiovascular and overall mortality, and measures of diet quality such as the HEI-2015 are becoming increasingly popular for assessing dietary intervention program impacts [52]. A recent systematic review of use of HEI-2015 for assessment of intervention impact within overweight/obese or high cardiometabolic risk adult populations offers insight into the use of this measure and considerations for interpretation. Notably, the authors emphasized that dietary interventions cannot be controlled with true placebos, and that contextual and cultural norms and changes in dietary patterns over time need to be considered, and therefore, a summary of diet quality as provided by indices such as the HEI-2015 should be thought of as promising “intermediate level” approaches to characterize diet when many food-behavior changes are being promoted [52]. The intervention studies included in the review demonstrated wide variation in magnitude of change that can be expected, providing evidence that the HEI-2015 may be useful but that further development is needed [52]. Therefore, despite null results for HEI-2015 in this analysis, future research should consider including HEI-2015 as a primary outcome for interventions targeting food-related behaviors. Including HEI-2015 as an outcome in future work will also contribute to the maintenance of current and up-to-date data as the HEI-2015 evolves to reflect changes to the DGA.

Finally, there was a statistically significant difference between Intervention and Comparison communities in baseline mean age, education, MSL score, and employment status. Although this analysis did not specifically investigate the potential reasons for these differences, it is possible that they could potentially impact diet quality, dietary intake, and other intervention outcomes. Also, as many of these factors are related to social determinants of health, it may have positioned participants in the Intervention communities to engage with the intervention in a different way than others might have. However, a separate analysis of sociodemographic and psychosocial factors associated with HEI-2015 scores in the preintervention evaluation sample of OPREVENT2 did not find any statistically significant relationship between HEI-2015 scores and these particular variables [38]. As a conservative approach, these variables were adjusted for in the statistical analyses. However, it is still worth noting that the baseline differences between the Intervention and Comparison communities exist and may have resulted in overly positive between-group effects that limited replication of findings.

### Strengths and limitations

This analysis had several strengths including a large sample size, high retention rate, and use of culturally adapted methods to modify and assess dietary intake. The dietary components of the intervention were developed and overseen by RDNs with continual input and feedback from each tribal community. This collaboration between RDNs, interventionists, and tribal members helped ensure the nutritional intervention component was grounded in the latest research for obesity prevention, culturally acceptable, and enhanced the likelihood of sustainability. Also, because the dietary component was delivered primarily in local food stores, the intervention has the potential to be scalable and transferable.

An additional strength was the communication of results back to individual participants and the communities. Participants were provided with a results card during anthropometric data collection that showed them their measurements and whether they were considered high risk according to established criteria. It was explained to participants that while the data collectors could not provide medical advice related to measurements taken, the participants could take the results card with them to their next medical appointment to discuss further with their providers. This was an important gesture to provide participants with potentially meaningful medical information but also to strengthen ties between the research team and the community members. Intervention progress and results have been and continue to be provided to tribal communities in quarterly and annual reports as well as informal presentations at board meetings.

There were also limitations to this analysis. These include the validity, reliability, and potential bias associated with the FFQ. FFQs have long been considered a valid and reliable assessment of dietary intake if properly culturally adapted and administered [30,32]. The FFQ used in this analysis was culturally adapted for and pilot tested in Native American populations. Despite this, it is possible that certain community specific foods were not adequately captured, and thus it is possible that the FFQ underestimated dietary intake in some instances. Furthermore, selection bias to enroll in the trial and social desirability to report healthful eating may have confounded the results.

Additional limitations include variability in data collection, seasonality, data collection bias, and exposure because of variability in implementation. Regarding data collection variability, most FFQs were printed and administered in English; however, some participants requested clarification and assistance in their native language. It is possible that certain details of the FFQs were lost through translation. With respect to seasonality, preintervention and postintervention data collection occurred during the same time of year, but each required multiple months to complete. Thus, it is possible that some data collection occurred during a change of seasons. Regarding data collection bias, data collectors were not blinded to which communities were allocated to Intervention and which were allocated to Comparison. This could have resulted in bias. Regarding variability in implementation, the process evaluation of OPREVENT2 found that dose, reach, and fidelity were variable among the different intervention components [34]. For example, the store component was implemented with high dose, reach, and fidelity while these same measures were reported as low-to-moderate for the school component. Although this is an expected limitation of MLMC interventions, it may nevertheless have contributed to variable exposure to each component reported by participants in Intervention communities, which may have influenced the intervention’s overall impact [53]. Finally, Native American populations represent incredibly diverse and culturally distinct tribal groups. While there are many similarities, there are also many important differences, which may impact generalizability of results to other Native American communities and individuals.

## Conclusions

This community-based MLMC intervention had significant impacts on individual intake of dietary fat and carbohydrates. These dietary behaviors are important key factors related to chronic disease risk. Our adjusted analysis supports the further implementation of MLMC interventions as one way to improve dietary intake among Native American populations post-colonization.

## Data Availability

Data described in the manuscript, code book, and analytic code will not be made publicly available because of agreements made with participating tribal communities.
